# Three-Arm Randomized Controlled Trial of Motivational Interviewing for Adolescent Drinking in Denmark

**DOI:** 10.1016/j.jaac.2026.07.009

**Published:** 2026-07-16

**Authors:** Lotte Vallentin-Holbech, Genevieve F. Dash, Sarah W. Feldstein Ewing, Anette Søgaard Nielsen, Janne S. Tolstrup, Kaare B. Wellnitz, Eva Ørnbøl, Kristine Rømer Thomsen

**Affiliations:** a Aarhus University, Aarhus, Denmark;; b University of Connecticut School of Medicine (Uconn Health), Farmington, Connecticut;; c University of Southern Denmark, Odense, Denmark;; d Psychiatric University Hospital, Odense, Region of Southern Denmark, Denmark;; e University of Southern Denmark, Copenhagen, Denmark;; f Aarhus University Hospital, Aarhus, Denmark

**Keywords:** adolescent, alcohol, motivational interviewing, randomized controlled trial, school

## Abstract

**Objective:**

To test the effectiveness of group motivational interviewing (GMI) and school/parent intervention (SP) in reducing drinking among public high school students in Denmark, a region with high rates of hazardous alcohol use.

**Method:**

This was a 3-arm, cluster-randomized controlled trial in 16 public high schools block-randomized to the following: (1) 2-session, manualized GMI coupled with SP; (2) SP-only; or (3) assessment-only control. A total of 2,766 students (65.53% female; mean age = 16.77 years, range = 15–19 years) completed online surveys during class periods at baseline and at 2, 6, 9, and 12 months. The primary outcome was past-month peak drinks per occasion at 12-month follow-up. Mixed models estimated change from baseline for each condition and mean change difference across conditions.

**Results:**

Students in the control condition significantly increased peak drinks over time at 2.5 times the rate of the other conditions (**Δ**mean = 1.13 [95% CI = 0.62, 1.64] vs 0.48 [95% CI = 0.03, 0.93] for GMI+SP and 0.47 [95% CI = 0.06, 0.88] for SP-only). The mean change difference in peak drinks for each GMI+SP and SP-only vs control was significant (**Δ**mean = −0.65 [95% CI = −1.28, −0.02] and −0.66 [95% CI = −1.27, −0.04], respectively), although significance did not persist after *p* value correction. More robust effects were observed for the secondary outcome of past-month total drinks.

**Conclusion:**

GMI coupled with school-based alcohol policies and parental education represents a promising approach to mitigating drinking among high school students. This study supports the crucial roles of developmentally salient relationships (eg, peers, parents/guardians) and contexts (eg, school, home) in developing and delivering intervention programs to adolescents.

**Clinical trial registration information:**

Prevention of Hazardous Use of Alcohol with Danish High School Students (Our Choice); https://clinicaltrials.gov/study/NCT06018389?cond=NCT06018389

Alcohol remains the most commonly used substance among adolescents around the world, with European adolescents evidencing some of the highest drinking rates.^1,2^ Although global rates of adolescent alcohol use have begun to decline, rates in Denmark have shown only modest declines compared to those in other Nordic countries^3^ and are among the highest across Europe, with 55% of 15- to 16-year-old adolescents reporting past-month heavy episodic drinking (HED; 5 or more drinks on a single drinking occasion).^4^ Alcohol use during this developmental window is of particular concern because it can increase the risk of persistent hazardous drinking,^5,6^ is associated with adverse structural and neurocognitive changes in the brain,^7,8^ and alcohol-related consequences experienced during adolescence can obstruct important developmental opportunities, diminish capacity to accomplish salient age-relevant tasks, and engender difficulties with effective functioning during the transition into adulthood.^9,10^ The sensitive nature of the adolescent developmental window, both behaviorally and neurodevelopmentally, underscores the critical need to deliver accessible and effective substance use prevention and intervention programs to this age group.

Motivational interviewing (MI)^11^ is an affirming, non-judgmental, and empathic approach that aims to explore and resolve ambivalence around drinking behavior, and remains one of the most robustly supported approaches for adolescent alcohol use.^12–16^ However, existing programs often show only modest effect sizes in adolescents and a subsequent return to baseline levels of substance use shortly after intervention delivery.^17^ One reason for this may be that most interventions for adolescents have been adapted from approaches originally developed for, and evaluated in, treatment-seeking adult populations. As a result, widely used interventions for adolescent substance use are not congruent with the developmental needs of the adolescent age group, and they do not leverage the unique characteristics of the adolescent developmental window, including the still-salient role of school contexts, in which highly influential peer factors are often embedded, and parents/guardians, with whom most adolescents still live.^9^ Given the high valence of peers during adolescence, group motivational interviewing (GMI) in particular can be highly engaging for this age group.^9,18^ Furthermore, schools represent an optimal “opportunistic setting” to offer timely and important intervention programs to otherwise difficult-to-reach youth. A recent pilot study of GMI for reducing alcohol use among high school students in Denmark supported its feasibility and acceptability, with students receiving GMI showing significant reductions in drinking compared to those in an assessment-only control condition.^19^

Multidimensional intervention approaches that target parents and school policies, in addition to individual-level and peer factors addressed more commonly in substance use interventions for adolescents (eg, GMI), have shown promise in reducing substance use, including in studies conducted in Europe, Asia, and the Americas.^13,20,21^ Parental involvement is a critically important component of prevention and intervention for this age group. In general, face-to-face interventions for adolescent substance use that include family/parent engagement are efficacious in reducing substance use,^22–24^ even within single-session formats.^25^ Although some high schools in Denmark have implemented school alcohol policies with the goal of lowering consumption, inconsistent enforcement has led to difficulty ascertaining the degree to which such policies affect student drinking, mirroring limitations in existing international studies. The effectiveness of comprehensive school alcohol policies on adolescent drinking thus remains unclear.^26,27^ However, given the salience of parents and the school environment in the lives of adolescents and their decisions around alcohol use, there is utility in integrating developmental considerations and salient stage-of-life factors into intervention programs for this age group.

## Present Study

Alcohol consumption is widely prevalent across all facets of adolescents’ lives in Denmark.^1,2^ Widespread drinking across family, peer, and social environments promotes normalization of alcohol use that, in turn, results in low perceived harms of drinking among adolescents and, oftentimes, their parents.^9,28^ This creates challenges in emphasizing alcohol use as a health concern. However, this cultural nuance also provides a unique opportunity to evaluate alcohol use interventions in a large sample of heavy-drinking adolescents, and in an ecologically valid environment. The present study aimed to evaluate the impact of school alcohol policy and parent education (SP), as well as the additive impact of GMI on several metrics of drinking behavior among students in Denmark over the course of their first year of high school. We hypothesized that students receiving GMI in addition to school alcohol policy and a parent meeting (GMI+SP) would show greater change in mean drinking, indicating reductions in alcohol use, as compared to students receiving only school alcohol policy and a parent meeting (SP-only) and compared to students receiving no additional intervention (assessment-only control). Adherence to and acceptability of the intervention was also evaluated.

## METHOD

This trial was registered at ClinicalTrials.gov (ID NCT06018389), the Danish Data Protection Agency, and followed the Consolidated Standards of Reporting Trials (CONSORT) 2010 guideline.^29,30^ Institutional review board approval was obtained from the Research Ethics Committee at Aarhus University, Denmark (ID BSS-2023–039). All elements of the study closely follow the published study protocol.^31^

### Participants

**FIGURE 1:**
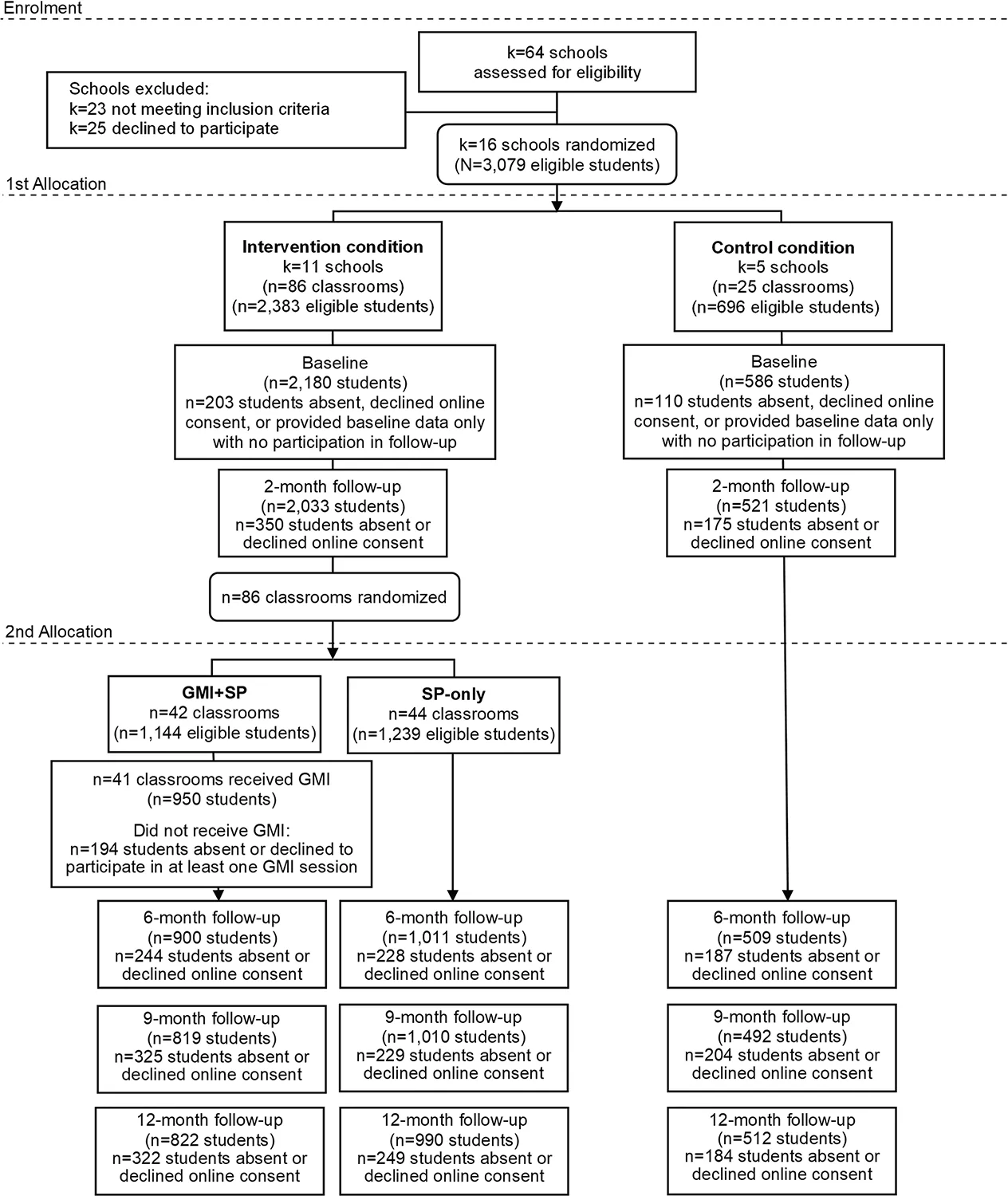
CONSORT Flow Diagram for Eligible Students (N = 3,079) **Note:** CONSORT = Consolidated Standards of Reporting Trials.

In spring 2023, a total of 64 public high schools were invited to participate in the study via email. Schools were eligible if they were available to participate during the school year of August 2023 to August 2024, did not have extensive alcohol prevention programs already in place, and agreed not to initiate any other substance use prevention programs during this period. A total of 16 eligible schools from 13 Danish cities fulfilled the criteria and agreed to participate. The schools include high schools in main cities of Denmark (3 of the 4 largest cities), as well as middle-sized and smaller cities, located in more rural areas. Eligibility criteria for students included being an incoming first-year high school student in August 2023, at least 15 years of age, able to understand and speak Danish, and able to give independent informed consent (the age of consent for research participation in Denmark is 15 years). A total of 3,079 eligible students were invited to participate via their school. The analytic sample included 2,766 students who provided consent to participate, were present in school the day that the baseline survey was administered, and provided data at baseline and a minimum of one of the follow-up surveys (65.53% female; mean age = 16.77 year, range = 15–19 years) (Table 1). Participant flow and retention rates are summarized in the CONSORT Flowchart (Figure 1).

### Procedures

Students and their parents or guardians received written information about the study at least 1 week before the study team visited campuses to conduct enrollment procedures. Information included that participation was voluntary and that students had the right to withdraw at any time. Students provided online consent ahead of the surveys, which were completed online, and written consent ahead of the first GMI session; consent from parents for participation in parent meetings was not required, per the university Ethics Committee. Student surveys were completed on-site in schools during class periods at 5 waves, namely, at baseline and at 2, 6, 9, and 12 months.

#### Trial Design and Randomization.

Randomization was conducted via 2 steps, as overseen by a third-party affiliate. Schools were randomized using uniformly random block sizes of 3 or 6 schools, with 66% (n = 11) of schools allocated to intervention and 33% (n = 5) allocated to assessment-only control. Subsequently, within schools randomized to intervention, classrooms were randomized at a 1:1 ratio to either GMI+SP or SP-only. We selected this randomization because randomizing to 3 arms at step 1 would have increased risk of bias via pre-existing differences between schools, despite reducing risk of cross-condition contamination within schools. In addition, it was not possible to randomize on the classroom level regarding schoolwide alcohol policy intervention, so we decided to randomize on classroom level to examine whether GMI yielded additive effects beyond SP while controlling by design for school-specific policy and parent education implementation. Randomization resulted in n = 42 classes (n = 1,144 eligible students) allocated to GMI+SP; n = 44 classes (n = 1,239 eligible students) allocated to SP-only; and n = 25 classes (n = 696 eligible students) allocated to assessment-only control. Randomization procedures are further detailed in the published study protocol.^31^ Schools randomized to assessment-only control were offered the interventions after completion of all data collection. Of the 5 control schools, 4 requested interventions, with 2 requesting both GMI+SP, 1 school requesting GMI-only, and 1 school requesting SP-only.

### Interventions

#### Group Motivational Interviewing.

GMI consisted of two 1-hour manualized sessions delivered 1 week apart. School classes were divided into mixed-gender groups of 6 to 9 students for delivery of sessions. Topics in the first session were “Tell your story,” “Gains from not drinking,” and “Social norms.” Topics in the second session were “Personal values,” “Linking values with behavior,” and “Planning/choices regarding alcohol.” All elements mirrored this team’s pilot trial.^19^ GMI sessions were delivered by trained study team members between administration of the 2- and 6-month follow-ups. The study was designed with this timeline because students start the school year in temporary, introductory classrooms before transitioning to permanent classrooms after 2 months. To ensure delivery within more stable peer groups, GMI was implemented shortly after students entered their permanent classrooms. School-level data collected at the 2-month follow-up were used to inform the social norms component of the GMI intervention.

#### School-Based Alcohol Policy.

Two months before the baseline assessment, schools assigned to active intervention participated in a 1-day workshop facilitated by the research team, during which school staff and student representatives (second- and third-year students) co-developed new initiatives, refined existing alcohol-related school policies, and subsequently agreed to implement this shared set of initiatives for the school year to reduce hazardous drinking at school-related events. The legal age for buying alcohol in Denmark is 16 years, although this restriction is rarely enforced, and it is very common for high schools to sell alcohol at parties and other school events. Newly implemented school-based alcohol policies included, but were not limited to, the following: increasing the number of alcohol-free school social events, promoting non-alcoholic beverages at school-based parties, working with student associations regarding their roles and responsibilities as role models, having more active parent involvement at school events, and actively enforcing the school’s alcohol policy. The school-based policy initiatives were implemented by school staff over the course of the academic year, commencing shortly after baseline. Most of the initiatives were implemented before the 6-month follow-up.

#### Parent Meeting.

Parents were invited to a 45-minute in-person meeting at the schools. In a 15-minute presentation, parents were shown local data on students’ alcohol use and its negative consequences in their immediate communities using data derived from the baseline survey within the school that their adolescent was attending. Parents were offered evidence-based parenting strategies, including, but not limited to the following: active participation and engagement in their adolescent’s life, monitoring of their adolescent’s activities in and out of school, clear communication about alcohol, and why parents may want to encourage their adolescent’s participation in organized activities (eg, sports). After the presentation, the study team facilitated a 30-minute discussion to have parents share experiences and strategies and to ask questions. Parents were given a resource sheet summarizing key points from the presentation. The parent session was delivered between administration of the baseline and the 2-month assessments, and we expected parents to have implemented potential changes at home before the 6-month follow-up.

#### Assessment-Only Control.

Students in the assessment-only control condition participated in the 5 data collection waves on the same schedule as those in the active intervention conditions. Schools in the control condition agreed not to initiate additional school-based alcohol policies or other new initiatives to prevent hazardous drinking during the 2023 to 2024 school year.

### Measures

#### Demographics.

Sample characteristics, including age, sex, and parent ethnicity were collected at baseline. Sex was indexed as sex assigned at birth (male/female), and parent ethnicity was indexed as number of Danish parents (both, one, none, unknown/missing).

#### Drinking Outcomes.

Past-month alcohol use was assessed using the Timeline Follow-Back (TLFB),^32^ a calendar-based assessment that prompts recall of drinking patterns by referencing dates, weekends, and notable events (eg, semester start or holidays). TLFB is widely used to assesses quantity and frequency of alcohol use in this age group, with the online version demonstrating comparable reliability to in-person formats.^33,34^ The TLFB included the primary outcome of past-month peak (maximum) drinks per drinking occasion at 12-month follow-up. It also assessed 2 secondary outcomes: total number of drinks consumed in the past month, and past-month heavy episodic drinking (HED) days (days drinking ≥5 drinks on 1 drinking occasion) at 12-month follow-up. Secondary outcomes also included changes in drinking behaviors at the 6- and 9-month follow-ups, after all interventions had been implemented. Change scores for each drinking outcome were calculated by subtracting the baseline score from the follow-up score, such that a negative change score value reflects reduced drinking and a positive change score value reflects increased drinking.

#### MI Fidelity and Acceptability.

The GMI intervention was manualized, and sessions were recorded for quality control by an affiliated fourth party who provided independent evaluation of MI fidelity. Randomly selected 20-minute segments of audio-recorded sessions (from 29 of 274 sessions) were coded by trained, independent coders using the Motivational Interviewing Treatment Integrity manual version 4.2.1 (MITI 4).^35^ Acceptability of GMI was assessed by asking participants whether they liked it, and whether they would recommend the intervention to next year’s students, using a 5-point Likert scale ranging from 1 = “I did not like it at all” to 5 = “I liked it a lot,” or 1 = “No, definitely not” to 5 = “Yes, definitely,” respectively.

#### Parent Meeting Acceptability.

Acceptability of the parent meeting was assessed by asking participating parents if they found the meeting relevant, using a 5-point Likert scale ranging from 1 = “Totally disagree” to 5 = “Totally agree” and whether they would recommend the intervention to next year’s parents (yes/no).

### Statistical Analysis

Power analyses are detailed extensively in this trial’s protocol paper.^31^ A sample size of 15 schools, with 2 to 11 participating classrooms for each school and class sizes ranging from 25 to 28 students, was required to achieve sufficient power of >0.80 for comparisons across all 3 conditions.

The present analyses were conducted in SAS version 9.4. As per protocol,^31^ analyses were conducted in an intent-to-treat sample comprising all students who participated in baseline data collection and a minimum of 1 follow-up survey. Primary hypotheses regarding relative effectiveness of each condition in changing alcohol use over time were evaluated using mixed models with restricted maximum likelihood estimation, which makes use of all available data even when some observations are incomplete. Unadjusted (base) models included condition, time, and their interaction. Adjusted (covariate) models also included baseline age (centered for analysis), sex, and parents’ ethnicity. All models used random intercepts to address baseline variability, an unstructured correlation matrix to account for within-subject correlations across repeated measurements, and robust standard errors to adjust for clustering within classroom and school.

**FIGURE 2:**
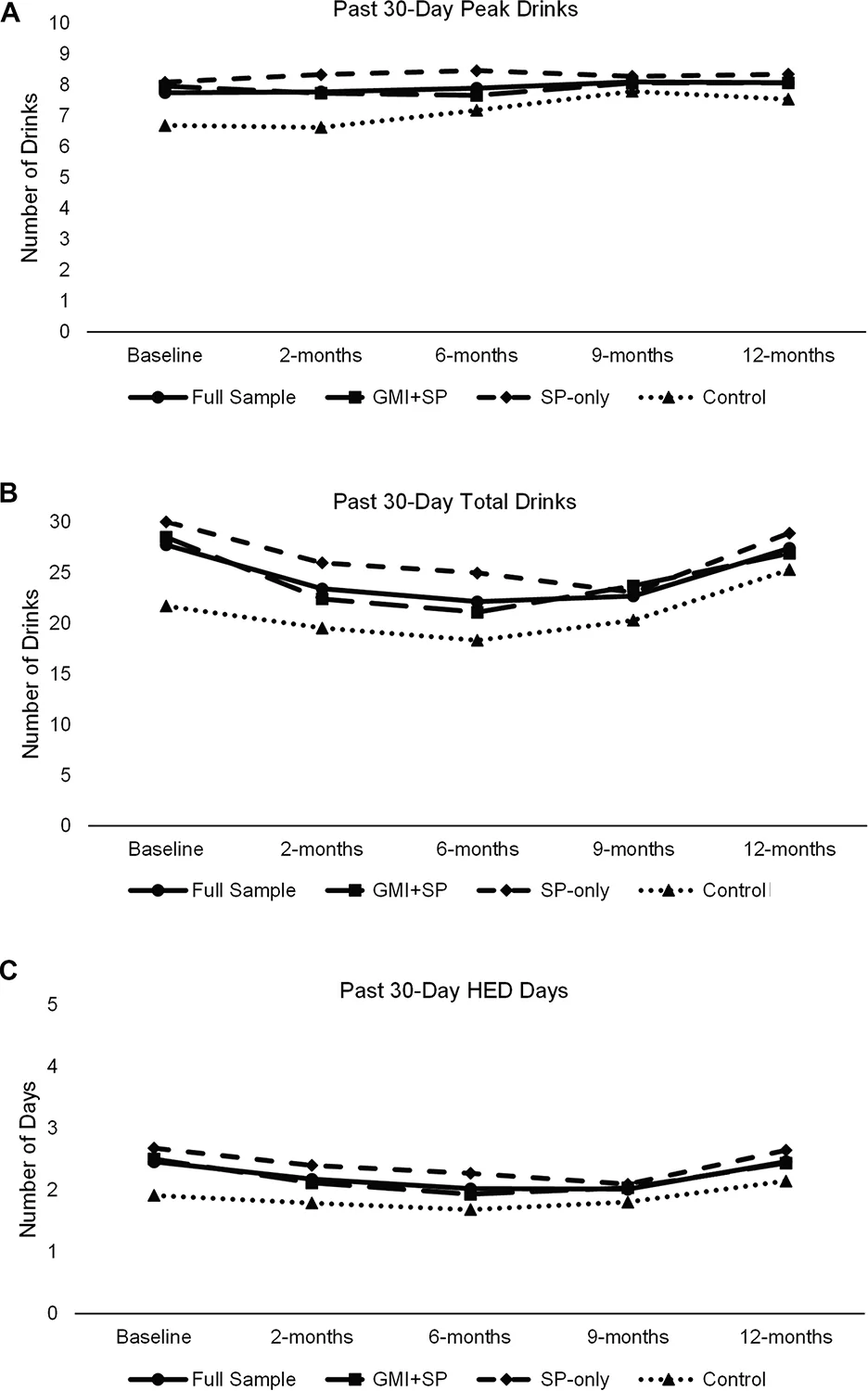
Raw Means for Past 30-Day Drinking Outcomes Across Condition and Measurement Occasion for (a) Peak Drinks, (b) Total Drinks, and (c) Heavy Episodic Drinking Days **Note:** GMI+SP = group motivational interviewing, school and parent intervention; HED = heavy episodic drinking; SP-only = school and parent intervention.

Inspection of the raw values (Figure 2) and raw change scores (Supplemental Table S1, available online) for each of the drinking outcome variables indicated the following: (1) a zero-inflated model, as planned in the protocol, was not needed because of a lower rate of zeros than anticipated and, in turn, a lack of preponderance of zeros that would necessitate such a model (in addition, this model was not appropriate for modeling change scores, which can take on negative values); and (2) linear change from baseline to 12-month follow-up was not a tenable assumption for these data. To account for this nonlinearity, analyses deviated slightly from the planned protocol. Mixed models were run with time modeled as a categorical variable, an approach that makes no assumption about the shape of change over time. Estimated marginal means (least-squares means) and pairwise comparisons of mean differences across conditions at each timepoint were generated using the *diff* command within the PROC GLIMMIX *lsmeans* statement. False discovery rate (FDR) adjustment was applied for *p* values from pairwise comparisons using the Benjamini–Hochberg procedure.^36^

## RESULTS

### Sample Characteristics and Descriptive Statistics

Table 1 displays descriptive statistics of the study variables at baseline. Students in the SP-only condition were, on average, slightly older (16.80 years) than students in the other 2 conditions (16.74–16.75 years). Students in the GMI+SP and SP-only conditions were more likely to have 2 Danish parents (74.01% and 74.06%, respectively) than students in the control condition (66.38%), and students in the control condition were more likely to have no Danish parents (12.46%) or unknown/missing (12.46%) than students in the GMI+SP (8.73% and 7.28%, respectively) and SP-only (8.87% and 7.06%, respectively) conditions. Although students in the control condition had lower levels of drinking at baseline compared to those in GMI+SP and SP-only, drinking levels were still high, with 72.18% reporting past-month drinking and averages of 6.62 peak drinks, 20.56 total drinks, and approximately 2 HED days. Retention rates within the analytic sample across the 4 follow-up waves were consistently high: 92.34% (n = 2,554) at 2 months, 87.49% (n = 2,420) at 6 months, 83.91% (n = 2,321) at 9 months, and 84.02% (n = 2,324) at 12 months.

### Differences in Drinking Outcomes Over Time

#### Primary Outcome: Peak Drinks in the Past Month.

In predicting peak drinks, there was a significant time by condition interaction in both unadjusted (*F*_6,6776_ = 2.69, *p* = .01) and adjusted (*F*_6,6775_ = 2.67, *p* = .01) models (Supplemental Table S2, available online). The primary outcome of interest was change in peak drinks between baseline and 12 months (as per pre-registration). After accounting for covariate effects, by 12 months, students in the control condition increased their reported peak drinks at a rate 2.5 times higher (**Δ**mean = 1.13, *p* < .0001) than students in the intervention conditions (GMI+SP: **Δ**mean = 0.48, *p* = .04; SP-only: **Δ**mean = 0.47, *p* = .03) (Table 2). Mean change differences for both GMI+SP and SP-only vs control were significant before, but not after, FDR correction (**Δ**mean difference = −0.65, *p* = .04, *p*_FDR_ = .08 and **Δ**mean difference = −0.66, *p* = .04, *p*_FDR_ = .08, respectively) (Table 3).

Secondary outcomes of interest were change between baseline and 6 months and change between baseline and 9 months. After accounting for covariate effects, students in the GMI+SP group maintained a steady level of peak drinks between baseline and 6 months (**Δ**mean = 0.09, *p* = .68), whereas moderate increases were observed in the SP-only (**Δ**mean = 0.54, *p* = .01) and control (**Δ**mean = 0.86, *p* = .0008) conditions (Table 2). The mean change difference for GMI+SP vs control was significant (**Δ**mean difference = −0.76, *p* = .02, *p*_FDR_ = .05) (Table 3). A similar pattern emerged between baseline and 9 months, wherein students in the GMI+SP group maintained a steady level of peak drinks between baseline and 9 months (**Δ**mean = 0.46, *p* = .054), whereas students in the SP-only and control conditions significantly increased (**Δ**mean = 0.49, *p* = .03 and **Δ**mean = 1.40, *p* < .0001, respectively) (Table 2). The mean change differences for both GMI+SP and SP-only vs control were significant (**Δ**mean difference = −0.94, *p* = .004, *p*_FDR_ = .02 and **Δ**mean difference = −0.91, *p* = .003, *p*_FDR_ = .02, respectively) (Table 3), indicating that students in the control group increased their peak drinks significantly more than students in the intervention conditions.

#### Secondary Outcome: Total Number of Drinks in the Past Month.

In predicting total drinks, there was a significant time by condition interaction in both unadjusted (*F*_6,6776_ = 2.59, *p* = .02) and adjusted (*F*_6,6775_ = 2.61, *p* = .02) models (Table S2, available online). After accounting for covariate effects, students in the control condition significantly increased their total drinks in the past 30 days between baseline and 12 months (**Δ**mean = 4.73, *p* = .0001), whereas students in the GMI+SP and SP-only groups remained stable (**Δ**mean = 0.99, *p* = .32 and **Δ**mean = 0.89, *p* = .37, respectively) (Table 2). Mean change differences for both GMI+SP and SP-only vs control were significant (**Δ**mean difference = −3.73, *p* = .01, *p*_FDR_ = .02 and **Δ**mean difference = −3.84, *p* = .008, *p*_FDR_ = .02, respectively) (Table 3).

As with peak drinks, we were also interested in changes between baseline and intermediate follow-up points for past-month total drinks. After accounting for covariate effects, students in the GMI+SP and SP-only groups significantly reduced their reported total drinks between baseline and 6 months (**Δ**mean = −5.58, *p* < .0001 and **Δ**mean = −3.17, *p* = .0006, respectively), whereas students in the control condition remained stable (**Δ**mean = −0.33, *p* = .74) (Table 2). The mean change differences between baseline and 6 months were significant across all group contrasts. The reduction observed for GMI+SP was greater than that for SP-only (**Δ**mean difference = −2.41, *p* = .03, *p*_FDR_ = .04), and for both GMI+SP and SP-only vs control (**Δ**mean difference = −5.24, *p* < .0001, *p*_FDR_ = .0005 and **Δ**mean difference = −2.31, *p* = .02, *p*_FDR_ = .03, respectively) (Table 3). Between baseline and 9 months, students in the GMI+SP and SP-only groups significantly reduced their reported total drinks (**Δ**mean = −4.32, *p* < .0001 and **Δ**mean = −4.61, *p* < .0001, respectively), whereas students in the control condition remained stable (**Δ**mean = 0.59, *p* = .58) (Table 2). Mean change differences for both GMI+SP and SP-only vs control were significant (**Δ**mean difference = −4.91, *p* = .0003, *p*_FDR_ = .0009 and **Δ**mean difference = −5.20, *p* < .0001, *p*_FDR_ = .0005, respectively) (Table 3).

#### Secondary Outcome: HED Days in the Past Month.

In predicting HED days, the time by condition was not significant in the unadjusted (*F*_6,6844_ = 1.78, *p* = .10) or adjusted (*F*_6,6843_ = 1.80, *p* = .10) model (Table S2, available online), indicating the absence of a global interaction effect (ie, across all conditions and timepoints). The following planned pairwise comparisons should be interpreted considering this, although such simple effects can detect significant local differences at specific time points that may appear even in the absence of a global effect. After accounting for covariate effects, students in the control condition significantly increased their HED days in the past 30 days between baseline and 12 months (**Δ**mean = 0.39, *p* = .002), whereas students in the GMI+SP and SP-only groups remained stable (**Δ**mean = 0.12, *p* = .21 and **Δ**mean = 0.07, *p* = .51, respectively) (Table 2). The mean change difference was significant for SP-only vs control participants (**Δ**mean difference = −0.32, *p* = .03, *p*_FDR_ = .05) (Table 3).

For the intermediate follow-ups, after accounting for covariate effects, students in both the GMI+SP and SP-only groups significantly reduced their reported HED days between baseline and 6 months (**Δ**mean = −0.40, *p* < .0001 and **Δ**mean = −0.28, *p* = .004, respectively) and between baseline and 9 months (**Δ**mean = −0.31, *p* = .004 and **Δ**mean = −0.44, *p* < .0001, respectively). Students in the control condition remained stable at both 6 months (**Δ**mean = −0.02, *p* = .85) and 9 months (**Δ**mean = 0.04, *p* = .73) (Table 2). The mean change differences for both GMI+SP and SP-only vs control were significant at both 6 months (**Δ**mean difference = −0.39, *p* = .002, *p*_FDR_ = .007 and **Δ**mean difference = −0.27, *p* = .03, *p*_FDR_ = .05, respectively) and 9 months (**Δ**mean difference = −0.35, *p* = .01, *p*_FDR_ = .04; **Δ**mean difference = −0.48, *p* = .0002, *p*_FDR_ = .002, respectively) (Table 3).

### Adherence and Acceptability

A total of 274 GMI sessions were delivered, achieving participation rates of 86.03% for the first session and 85.79% for the second; 77.68% of students participated in both sessions. GMI leaders received fair ratings in all MITI categories and demonstrated high (>85%) adherence to the GMI manual. In total, 76.78% of the participating students liked the GMI sessions; 2.66% reported not liking the GMI sessions; and 20.56% were neutral. Similarly, 75.38% reported that they would recommend that high schools offer the GMI sessions to next year’s students; 5.84% reported that they would not recommend this; and 18.78% were neutral.

Approximately 1,953 parents participated in the parent meetings, representing 2,383 students, and 1,138 parents responded to the brief anonymous survey on acceptability. Some responses were provided jointly by both caregivers of the same student, as caregivers were allowed to discuss and complete the survey together. Consequently, the number of responses likely underestimate the total number of participating parents. Of these, 81.28% found the meeting relevant, and 92.09% recommended that the high school invite new parents next year.

## DISCUSSION

The aim of this study was to compare GMI in combination with school-based alcohol policies and a parent meeting (GMI+SP) to SP-only and assessment-only control, as a means of determining the comparative degrees of behavior change in drinking in the first year of high school in Denmark, a country with high rates of hazardous alcohol use among adolescents.^1,2^ The primary outcome was peak drinks per drinking occasion at 12 months, and secondary outcomes were past-month total number of drinks and number of HED days at 12 months. Secondary outcomes also included changes in drinking behaviors at 6- and 9-month follow-ups, where all interventions had been implemented.

Although students across conditions increased peak drinks between baseline and 12 months, students in the control condition significantly increased peak drinks at 2.5 times the rate of those in the intervention conditions, indicating a preventive effect of GMI+SP and SP-only. This preventive effect was further demonstrated by a significant decrease in total drinks and HED days at 6 and 9 months, and the absence of a significant increase between baseline and 12 months. A consistent pattern across the drinking metrics was the attenuation of intervention effects in GMI+SP and SP-only by the 12-month follow-up, which is aligned with past studies on long-term outcomes of MI interventions for adolescent drinking.^15^ Such a pattern suggests that biannual delivery of prevention programs, including “booster” sessions for students who have participated in the programs, may be indicated. This attenuation may also be partly attributed to contextual factors, as past-month measures here partly covered students’ summer holiday, a period typically associated with higher alcohol use among adolescents and with less influence of school alcohol policies.^37^ Notably, GMI+SP and SP-only outperformed control on most drinking metrics at most timepoints, and students in the control condition showed no significant reductions in drinking at any point while evidencing significant increases on all drinking metrics by 12 months. These patterns are consistent with existing literature showing an increase in adolescent drinking over time in the absence of prevention programming.^28,38,39^

In considering these findings, it is worthwhile to note the following: (1) the magnitude of effect in comparing GMI+SP and SP-only to control was relatively small, and (2) GMI+SP did not outperform SP-only, with some exceptions. Indeed, meta-analytic evidence indicates small overall effects on alcohol outcomes, albeit with stronger effects for heavier drinking indicators.^20,40^ These patterns are consistent with intervention studies more broadly, in which effect sizes are often small and significant differences between active intervention conditions are difficult to detect.^41^ Although combining GMI with a school and parent component did not yield consistent additive benefits over SP-only, the GMI+SP condition appeared to confer some advantage for peak drinks and total drinks at 6 months; in addition, peak drinks remained stable in the GMI+SP condition at 9 months, while increasing in the SP-only condition. This is consistent with findings that MI-based group processes can influence high-risk drinking, potentially through increased motivation and peer-related change processes.^19,21^ Taken together, these findings suggest that school-, parent-, and student-focused components may operate through complementary pathways: the school-parent intervention constraining overall escalation, and group MI targeting more acute risk behaviors.^42,43^ Consistent with the broader literature, multi-component approaches do not consistently outperform single-component programs,^20^ highlighting the need for further research on how and when such components produce synergistic effects.

Parent meetings and GMI sessions had high participation rates and were rated highly positive by students and parents, and the quality of GMI facilitation was rated as fair, highlighting the acceptability and feasibility of these interventions. The degree of openness and engagement by these schools, which are notably overwhelmed public high schools, indicate 3 critical findings. First, and most important, is the need for identifying prevention and intervention programs for alcohol use in this age group, when there is a normative surge in alcohol use. This pattern was evidenced by the control schools, whose students showed an acceleration of drinking across all measured drinking behaviors from baseline to 12 months in the absence of GMI or SP interventions. Second, it indicates that these 2 different types of interventions (school-based policies and parental engagement, and active behavioral intervention, such as GMI) were tenable approaches in these public-school settings. Third, the preliminary findings for GMI+SP indicate a potential additive effect of such group-based approaches, which should be examined in future studies including designs with limited risk of contamination between conditions. Beyond these points, it is important to note that the GMI and SP interventions were deliberately developed to be accessible and relevant to students across diverse backgrounds, emphasizing equity in access to services. Our team’s interventions are designed to be universal and easily implemented into opportunistic settings (eg, schools) at low cost; the interventions require no licensing and contain no proprietary materials, meaning that the interventionists’ time is the only cost associated with delivering the intervention. Next steps will include evaluating such a school-based approach in a more diverse sample, for example, in the United States.

Findings should be interpreted in light of limitations. First, although overall retention was high throughout the 12-month study period, some attrition occurred, as is typical in longitudinal trials. Second, despite random assignment, baseline differences were observed across conditions. Although covariate adjustment and implicit inclusion of baseline scores via use of change scores were used to address these imbalances, such methods cannot fully eliminate potential bias. Third, randomizing classrooms to GMI+SP or SP-only within the same schools introduces some risk of contamination, which may have limited potential effects of GMI+SP vs SP-only.

This study represents one of the first trials to evaluate the effectiveness of GMI, school-level policies, and parent-level interventions aimed at reducing drinking among high school students. Findings suggest that these intervention strategies, particularly the condition leveraging GMI, school policy, and parental intervention, were effective in reducing several metrics of alcohol use up to 9 months after first assessment and preventing increases in drinking up to 12 months after first assessment. Shifts observed by the 12-month follow-up indicate the potential benefit of annual booster interventions to sustain the positive impact on students’ drinking behavior. Future research should explore subgroup effects and seasonal drinking patterns. These findings offer novel insights into multi-level prevention strategies targeting adolescent alcohol use, and can help inform evidence-based alcohol prevention policies, educational strategies, and professional practice in promoting adolescent health.

## Supplementary Material

Supplement

## Figures and Tables

**TABLE 1:** Sample Characteristics at Baseline

Variable	Full sample (N = 2,766)		GMI+SP (n = 962)		SP-only (n = 1,218)		Control (n = 586)		Difference test	
Demographics	Mean	SD	Mean	SD	Mean	SD	Mean	SD	*F*(2)	*p*
Age, y	16.77	0.58	16.75	0.55	16.80	0.58	16.74	0.62	3.51	.03
Gender	**N**	**%**	**n**	**%**	**n**	**%**	**n**	**%**	**χ^2^(2)**	***p***
Female	1,812	65.53	634	65.90	789	64.83	389	66.38	0.51	.77
Male	953	34.47	328	34.10	428	35.17	197	33.62		
Unknown/missing	1	0.00	0	0.00	1	0.00	0	0.00		
Parents’ ethnicity	**N**	**%**	**n**	**%**	**n**	**%**	**n**	**%**	**χ^2^(6)**	***p***
Both Danish	2,003	72.42	712	74.01	902	74.06	389	66.38	26.68	.0002
One Danish	269	9.73	96	9.98	122	10.02	51	8.70		
Neither Danish	265	9.58	84	8.73	108	8.87	73	12.46		
Unknown/missing	229	8.28	70	7.28	86	7.06	73	12.46		
Drinking history	**N**	**%**	**n**	**%**	**n**	**%**	**n**	**%**	**χ^2^(2)**	***p***
Lifetime drinking	2,454	88.72	864	89.81	1,096	89.98	494	84.30	14.52	.0007
Past–30-day drinking	2,190	79.18	777	80.77	990	81.28	423	72.18	22.13	<.0001
Primary outcome	**Mean**	**SD**	**Mean**	**SD**	**Mean**	**SD**	**Mean**	**SD**	***F*(2)**	***p***
Past–30-day peak drinks	7.77	6.72	7.95	6.74	8.18	6.73	6.62	6.57	11.61	<.0001
Secondary outcomes	**Mean**	**SD**	**Mean**	**SD**	**Mean**	**SD**	**Mean**	**SD**	***F*(2)**	***p***
Past–30-day total drinks	27.16	31.58	28.05	31.46	29.65	32.97	20.56	27.73	20.13	<.0001
Past–30-day HED days	2.45	2.88	2.50	2.85	2.71	3.03	1.84	2.50	21.59	<.0001

**Note:** Difference tests were conducted using Welch analysis of variance for continuous variables and **χ**^2^ test for categorical variables. Boldface type denotes statistical significance. GMI = group motivational interviewing; HED = heavy episodic drinking; SP = school and parent intervention.

**TABLE 2:** Predicted Mean Change from Baseline Within Each Condition at Follow-up Points

	Unadjusted models					
Outcome	6 mo		9 mo		12 mo	
Peak drinks	Estimate	[95% CI], *p*	Estimate	[95% CI], *p*	Estimate	[95% CI], *p*
GMI + SP	−0.24	[−0.61, 0.14], .22	0.13	[−0.28, 0.54], .53	0.15	[−0.24, 0.54], .45
SP-only	0.18	[−0.19, 0.55], .33	0.14	[−0.24, 0.52], .46	0.12	[−0.25, 0.48], .54
Control	0.58	[0.09, 1.06], .02	1.12	[0.64, 1.60], <.0001	0.85	[0.36, 1.34], .0007
Total drinks	Estimate	[95% CI], *p*	Estimate	[95% CI], *p*	Estimate	[95% CI], *p*
GMI + SP	−7.44	[−9.06, −5.81], <.0001	−6.19	[−7.96, −4.42], <.0001	−0.87	[−2.62, 0.88], .33
SP-only	−5.17	[−6.69, −3.65], <.0001	−6.60	[−8.14, −5.06], <.0001	− 1.13	[−2.78, 0.52], .18
Control	−1.92	[−3.77, −0.06], .04	−1.02	[−3.01, 0.97], .32	3.13	[0.85, 5.41], .007
HED days	Estimate	[95% CI], *p*	Estimate	[95% CI], *p*	Estimate	[95% CI], *p*
GMI + SP	−0.55	[−0.71, −0.29], <.0001	−0.46	[−0.64, −0.27], <.0001	−0.02	[−0.19, 0.15], .29
SP-only	−0.44	[−0.60, −0.29], <.0001	−0.60	[−0.74, −0.45], <.0001	−0.09	[−0.26, 0.08], .81
Control	−0.14	[−0.31, 0.04], .12	−0.09	[−0.29, 0.11], .41	0.27	[0.04, 0.50], .02
	Adjusted models					
Outcome	6 mo		9 mo		12 mo	
Peak drinks	Estimate	[95% CI], *p*	Estimate	[95% CI], *p*	Estimate	[95% CI], *p*
GMI + SP	0.09	[−0.35, 0.53], .68	0.46	[−0.01, 0.93], .05	0.48	[0.03, 0.93], .04
SP-only	0.54	[0.11, 0.96], .01	0.49	[0.06, 0.92], .03	0.47	[0.06, 0.88], .03
Control	0.86	[0.36, 1.36], .0008	1.40	[0.90, 1.91], <.0001	1.13	[0.62, 1.64], <.0001
Total drinks	Estimate	[95% CI], *p*	Estimate	[95% CI], *p*	Estimate	[95% CI], *p*
GMI + SP	−5.58	[−7.45, −3.70], <.0001	−4.32	[−6.34, −2.29], <.0001	0.99	[−0.97, 2.96], .32
SP-only	−3.17	[−4.98, −1.35], .0006	−4.61	[−6.45, −2.78], <.0001	0.89	[−1.05, 2.83], .37
Control	−0.33	[−2.28, 1.62], .74	0.59	[−1.52, 2.70], .58	4.73	[2.31, 7.14], .0001
HED days	Estimate	[95% CI], *p*	Estimate	[95% CI], *p*	Estimate	[95% CI], *p*
GMI + SP	−0.40	[−0.59, −0.21], <.0001	−0.31	[−0.53, −0.10], .004	0.12	[−0.07, 0.31], .21
SP-only	−0.28	[−0.48, −0.09], .004	−0.44	[−0.63, −0.26], <.0001	0.07	[−0.14, 0.27], .51
Control	−0.02	[−0.21, 0.17], .85	0.04	[−0.18, 0.26], .73	0.39	[0.14, 0.65], .002

**Note:** Unadjusted models include condition, time, and their interaction; adjusted models include covariates (baseline age, gender, parents’ ethnicity). Boldface type denotes significant difference from 0 (where 0 denotes no change from baseline); GMI = group motivational interviewing; HED = heavy episodic drinking; SP = school and parent intervention.

**TABLE 3:** Mean Change Difference Comparisons Across Conditions at Each Follow-up Point (Adjusted Models)

Peak drinks Time	Group 1	Group 2 (ref)	Estimate [95% CI]	*T* df = 6775	*p*	FDR *p*
Baseline to 6 mo	GMI + SP	SP-only	−0.44 [−0.97, 0.09]	−1.64	.10	.15
	SP-only	Control	−0.32 [−0.93, 0.29]	−1.03	.30	.39
	GMI + SP	Control	−0.76 [−1.38, −0.15]	−2.43	.02	.05
Baseline to 9 mo	GMI + SP	SP-only	−0.03 [−0.59, 0.53]	−0.10	.92	.98
	SP-only	Control	−0.91 [−1.52, −0.30]	−2.94	.003	.02
	GMI + SP	Control	−0.94 [−1.58, −0.31]	−2.92	.004	.02
Baseline to 12 mo	GMI + SP	SP-only	0.01 [−0.53, 0.54]	0.02	.98	.98
	SP-only	Control	−0.66 [−1.27, −0.04]*	−2.09	.04*	.08
	GMI + SP	Control	−0.65 [−1.28, −0.02]*	−2.02	.04*	.08
Total drinks Time	Group 1	Group 2 (ref)	Estimate [95% CI]	*T* df = 6776	*p*	FDR *p*
Baseline to 6 mo	GMI + SP	SP-only	−2.41 [−4.62, −0.20]	−2.14	.03	.04
	SP-only	Control	−2.31 [−5.24, −0.43]	−2.31	.02	.03
	GMI + SP	Control	−5.24 [−7.71, −2.77]	−4.16	<.0001	.0005
Baseline to 9 mo	GMI + SP	SP-only	0.30 [−2.04, 2.63]	0.25	.80	.90
	SP-only	Control	−5.20 [−7.72, −2.69]	−4.06	<.0001	.0005
	GMI + SP	Control	−4.91 [−7.57, −2.25]	−3.61	.0003	.0009
Baseline to 12 mo	GMI + SP	SP-only	0.11 [2.30, 2.52]	0.09	.93	.93
	SP-only	Control	−3.84 [−6.68, −1.00]	−2.65	.008	.02
	GMI + SP	Control	−3.73 [−6.62, −0.84]	−2.53	.01	.02
HED days Time	Group 1	Group 2 (ref)	Estimate [95% CI]	*T* df = 6843	*p*	FDR *p*
Baseline to 6 mo	GMI + SP	SP-only	−0.12 [−0.34, 0.10]	−1.06	.29	.33
	SP-only	Control	−0.27 [−0.50, −0.03]	−2.20	.03	.05
	GMI + SP	Control	−0.39 [−0.63, −0.15]	−3.17	.002	.007
Baseline to 9 mo	GMI + SP	SP-only	0.13 [−0.11, 0.36]	1.05	.29	.33
	SP-only	Control	−0.48 [−0.72, −0.21]	−3.69	.0002	.002
	GMI + SP	Control	−0.35 [−0.62, −0.06]	−2.49	.01	.04
Baseline to 12 mo	GMI + SP	SP-only	0.05 [−0.18, 0.29]	0.44	.66	.66
	SP-only	Control	−0.32 [−0.61, −0.04]	−2.20	.03	.05
	GMI + SP	Control	−0.27 [−0.56, 0.02]	−1.84	.07	.10

**Note:** Adjusted models include covariates (baseline age, gender, parents’ ethnicity). Boldface type denotes statistical significance after FDR correction. FDR = false discovery rate; GMI = group motivational interviewing; HED = heavy episodic drinking; SP = school and parent intervention.

* Denotes statistical significance before, but not after, FDR correction.

## Data Availability

The data that support the findings of this study are available from the corresponding author upon reasonable request.
