# Supplementary material for: Three-Arm Randomized Controlled Trial of Motivational Interviewing for Adolescent Drinking in Denmark
Source: J Am Acad Child Adolesc Psychiatry. Author manuscript; Available in PMC 2026 Sep 11. (PMC13562902; doi:10.1016/j.jaac.2026.07.009)
Supplement: Supplementary file 1 [file NIHMS2205839-supplement-Supplement.docx]

**Supplemental Materials**

**For**

**Three-arm randomized controlled trial of motivational interviewing for adolescent drinking in Denmark**

Lotte Vallentin-Holbech, PhD,^a^ Genevieve F. Dash, PhD,^a,b^ Sarah W. Feldstein Ewing, PhD,^a,b^ Anette Søgaard Nielsen, PhD,^c,d^ Janne Tolstrup, PhD,^e^ Kaare Bro Wellnitz, PhD,^a,f^ Eva Ørnbøl, PhD,^f^ & Kristine Rømer Thomsen, PhD^a*^

^a^Aarhus University, Aarhus, Denmark

^b^University of Connecticut School of Medicine, Farmington, Connecticut, USA

^c^University of Southern Denmark, Odense, Denmark,

^c^Psychiatric University Hospital, Odense, Region of Southern Denmark, Denmark

^e^University of Southern Denmark, Copenhagen, Denmark

^f^Aarhus University Hospital, Aarhus, Denmark

*Correspondence concerning this article should be directed to Prof. Kristine Rømer Thomsen, Centre for Alcohol and Drug Research, Aarhus BSS, Aarhus University, Bartholins Allé 10, building 1322, DK-8000 Aarhus, Denmark, email: [krt.crf@psy.au.dk](mailto:krt.crf@psy.au.dk)

| **Table S1: Raw Change Scores Between Baseline and Follow-Up for Alcohol Use Outcomes in the Full Sample and by Condition** | | | | | | | | |
| --- | --- | --- | --- | --- | --- | --- | --- | --- |
| Change from Baseline | Full Sample | | GMI+SP | | SP-Only | | Control | |
|  | Mean | SD | Mean | SD | Mean | SD | Mean | SD |
| Peak Drinks |  |  |  |  |  |  |  |  |
| 6-Months | 0.16 | 5.96 | -0.24 | 5.84 | 0.22 | 6.19 | 0.73 | 5.69 |
| 9-Months | 0.34 | 6.12 | 0.11 | 6.18 | 0.14 | 6.27 | 1.14 | 5.64 |
| 12-Months | 0.30 | 5.90 | 0.15 | 5.84 | 0.16 | 5.99 | 0.79 | 5.79 |
| Total Drinks |  |  |  |  |  |  |  |  |
| 6-Months | -5.27 | 24.73 | -7.50 | 25.12 | -5.05 | 25.34 | -1.74 | 22.31 |
| 9-Months | -5.33 | 25.65 | -6.24 | 26.74 | -6.60 | 25.59 | -1.23 | 23.51 |
| 12-Months | -0.21 | 26.54 | -1.05 | 26.33 | -1.18 | 26.57 | 3.04 | 26.63 |
| HED Days |  |  |  |  |  |  |  |  |
| 6-Months | -0.42 | 2.48 | -0.58 | 2.48 | -0.44 | 2.62 | -0.13 | 2.11 |
| 9-Months | -0.43 | 2.59 | -0.43 | 2.80 | -0.60 | 2.50 | -0.09 | 2.37 |
| 12-Months | 0.01 | 2.65 | -0.02 | 2.50 | -0.11 | 2.72 | 0.27 | 2.73 |
| **Note:** Change score reflects baseline score subtracted from score at a given follow-up point; GMI = group motivational interviewing; SP = school and parent intervention. | | | | | | | | |

| **Table S2: Tests of Fixed Effects from Models Predicting Change in Past 30-day Drinking Outcomes** | | | | | | |
| --- | --- | --- | --- | --- | --- | --- |
|  | Peak Drinks | | | | | |
|  | Unadjusted Model | | | Adjusted Model | | |
| Predictor | F | df | p | F | df | p |
| Condition | **4.22** | **2, 6776** | **.01** | **3.62** | **2, 6775** | **.03** |
| Time | **5.14** | **3, 6776** | **.002** | **4.87** | **3, 6775** | **.002** |
| Time x Condition | **2.69** | **6, 6776** | **.01** | **2.67** | **6, 6775** | **.01** |
| Age | - | - | - | **8.03** | **1, 6775** | **.005** |
| Sex | - | - | - | 3.48 | 1, 6775 | .06 |
| Ethnicity | - | - | - | 2.51 | 3, 6775 | .06 |
|  | Total Drinks | | | | | |
|  | Unadjusted Model | | | Adjusted Model | | |
| Predictor | F | df | p | F | df | p |
| Condition | **10.51** | **2, 6776** | **<.0001** | **9.01** | **2, 6775** | **.0001** |
| Time | **39.25** | **3, 6776** | **<.0001** | **39.40** | **3, 6775** | **<.0001** |
| Time x Condition | **2.59** | **6, 6776** | **.02** | **2.61** | **6, 6775** | **.02** |
| Age | - | - | - | **14.64** | **1, 6775** | **.0001** |
| Sex | - | - | - | **7.78** | **1, 6775** | **.005** |
| Ethnicity | - | - | - | **5.11** | **3, 6775** | **.002** |
|  | Heavy Episodic Drinking Days | | | | | |
|  | Unadjusted Model | | | Adjusted Model | | |
| Predictor | F | df | p | F | df | p |
| Condition | **7.30** | **2, 6844** | **.007** | **6.03** | **2, 6843** | **.002** |
| Time | **27.68** | **3, 6844** | **<.0001** | **27.83** | **3, 6843** | **<.0001** |
| Time x Condition | 1.78 | 6, 6844 | .10 | 1.80 | 6, 6843 | .10 |
| Age | - | - | - | **14.99** | **1, 6843** | **.0001** |
| Sex | - | - | - | **6.61** | **1, 6843** | **.01** |
| Ethnicity | - | - | - | 2.42 | 3, 6843 | .06 |
